# Fibrosis score predicts mortality in patients with fibrotic hypersensitivity pneumonitis

**DOI:** 10.3389/fmed.2023.1131070

**Published:** 2023-03-21

**Authors:** Ju Hyun Oh, Jieun Kang, Jin Woo Song

**Affiliations:** ^1^Department of Pulmonary and Critical Care Medicine, Sanggye Paik Hospital, Inje University College of Medicine, Seoul, Republic of Korea; ^2^Division of Pulmonary and Critical Care Medicine, Department of Internal Medicine, Ilsan Paik Hospital, Inje University College of Medicine, Goyang-si, Gyeonggi-do, Republic of Korea; ^3^Department of Pulmonary and Critical Care Medicine, Asan Medical Center, University of Ulsan College of Medicine, Seoul, Republic of Korea

**Keywords:** hypersensitivity pneumonitis, mortality, prognosis, fibrosis, high-resolution computed tomography

## Abstract

**Background:**

Variable clinical courses make it challenging to predict mortality resulting from fibrotic hypersensitivity pneumonitis (HP). This study evaluated the usefulness of radiologic parameters for predicting mortality in patients with fibrotic HP.

**Methods:**

Clinical data and high-resolution computed tomography (HRCT) images, which were scored for reticulation, honeycombing, ground glass opacity (GGO), consolidation, and mosaic attenuation (MA) by visual assessment, were retrospectively analyzed in a total of 101 patients with fibrotic HP (all biopsy-proven cases). Fibrosis score was defined as the sum of reticulation and honeycombing scores.

**Results:**

The mean age of the 101 patients was 58.9 years, and 60.4% were females. During the follow-up (median: 55.5 months; interquartile range: 37.7–89.0 months), the 1-, 3-and 5-year mortality rates were 3.9, 16.8, and 32.7%, respectively. The non-survivors were older and had significantly lower lung function and minimum oxygen saturation during the 6-min walk test than the survivors. The non-survivors had higher scores of reticulation, honeycombing, GGO, fibrosis, and MA on HRCT than survivors. In the multivariable Cox analysis, reticulation, GGO, and fibrosis scores were independent prognostic factors for mortality in patients with fibrotic HP, as well as age. Fibrosis score showed great performance for predicting the 5-year mortality (AUC = 0.752, *p* < 0.001) and higher mortality was recorded for patients with high fibrosis score (≥12.0%) (the mean survival time: 58.3 vs. 146.7 months, *p* < 0.01) than those without.

**Conclusion:**

Our results suggest that radiologic fibrosis score may be a useful predictor of mortality in patients with fibrotic HP.

## Introduction

Hypersensitivity pneumonitis (HP) is a diffuse interstitial lung disease (ILD) caused by an immune response to inhaled antigens in susceptible individuals (1, 2). Historically, HP was categorized into subtypes as acute, subacute, or chronic based on the disease duration. In general, acute HP has favorable outcome by avoiding exposure to the suspected antigen. However, in the case of subacute or chronic HP, it has been challenging for clinicians to predict the prognosis due to variable disease courses.

Previous studies have shown that several factors are associated with prognosis in chronic HP (3–6). For example, smoking history, unknown inciting antigens, decline in forced vital capacity (FVC) and fibrotic nonspecific interstitial pneumonia pattern or usual interstitial pneumonia (UIP) pattern on histologic examination, have been identified as significant predictors of poor prognosis in patients with chronic HP (5, 7–11). Furthermore, HP patients with radiological fibrosis were reported to have worse survival outcomes than those without (4, 12, 13); Chung et al. showed that of 132 patients with chronic HP, those with reticulation or honeycombing on high-resolution computed tomography (HRCT) had significantly worse survival than those without (*p* = 0.016 or 0.007 in log-rank test, respectively) (4). Hanak et al. also reported that in 69 patients with chronic HP, subjects with fibrosis on HRCT had a higher mortality rate than those without (42% vs. 2%, *p* < 0.001) (14). As such, it has become important to determine the presence of fibrosis in predicting prognosis for patients with HP. Given these points, recent guidelines for diagnosing HP suggest that to better reflect clinical manifestation and courses, HP can be classified as fibrotic or non-fibrotic depending on the radiologic or histopathologic evidence of pulmonary fibrosis (1, 15). However, in patients with fibrotic HP, the prognosis is difficult to predict due to diverse courses, and the role of radiological parameters are not well defined. Therefore, in this study, the role of radiological parameters in predicting mortality was investigated through the quantification of several radiological findings identified in fibrotic HP.

## Methods

### Study population

From January 2002 to December 2017, 106 patients suspected of fibrotic HP were identified at Asan Medical Center, Seoul, Republic of Korea. Among them, patients with lack of histopathological confirmation (*n* = 5) were excluded, and 101 patients diagnosed with fibrotic HP by histopathologic findings obtained *via* surgical (*n* = 99) or transbronchial lung biopsy (*n* = 2) were included in this study. All diagnoses were made through multidisciplinary discussion. All patients were included in previous studies (12, 16, 17). The diagnosis of HP was reconfirmed by multidisciplinary discussion based on the criteria of the American Thoracic Society (ATS), Japanese Respiratory Society (JRS), and Latin American Thoracic Association (ALAT) (1). This study was approved by the Institutional Review Board of Asan Medical Center (2017–1081), and the requirement for informed consent was waived due to the study’s retrospective nature.

### Clinical data

Clinical and survival data from all patients were retrospectively collected using medical records and/or the records from the National Health Insurance of Korea. A detailed history of exposure to various environmental factors known to induce HP was obtained from all participants at the time of initial diagnosis *via* questionnaires. FVC by spirometry, total lung capacity (TLC) by plethysmography and diffusing capacity of the lung for carbon monoxide (DLco) were measured according to the ATS/ERS recommendations (18–23). The results were expressed as a percentage of predicted values. The 6-min walk test (6MWT) was performed following the guidelines (24). The ILD-GAP index was calculated based on the type of ILD and clinical variables (gender, age, FVC, and DLco), as suggested previously (25). The time of diagnosis was defined as the date on which the lung biopsy was performed. Baseline clinical data including lung function and 6MWT were obtained at the time of diagnosis.

### HRCT images

All HRCT scans were performed at the time of diagnosis. As described in our previous study (17), HRCT images were assessed by two dedicated thoracic radiologists (4 and 17 years of experience, respectively), blinded to the patient’s clinical information, and semi-quantitatively scored for reticulation, honeycombing, ground glass opacity (GGO), consolidation, and mosaic attenuation (MA) in six lung zones to the nearest 10^th^ percentile. Fibrosis score was defined as the sum of reticulation and honeycombing scores (26). Level of inter-reader agreement of the HRCT images was evaluated using Cohen’s kappa. Inter-reader agreement in assessing HRCT patterns showed moderate strength of agreement (*κ* = 0.761; 95% confidence interval [CI], 0.634–0.888) (27). Discrepancies were resolved by a consensus.

HRCT findings were classified as a UIP-like pattern or not; a UIP-like pattern was defined as an HRCT pattern compatible with a UIP or probable UIP pattern according to 2018 Fleischner Society IPF diagnostic guidelines, and some modifications were made to apply to HP (28, 29). Briefly, a UIP-like pattern was defined as reticulation with traction bronchiectasis with/without honeycombing in the absence of features to suggest an alternative diagnosis such as peribronchovascular predominance with subpleural sparing, predominant consolidation, extensive pure GGO or diffuse nodules/cysts; however, presence of MA and distribution of fibrosis were not included in the features of an alternative diagnosis.

### Statistical analysis

All values are expressed as mean ± standard deviation for continuous variables or numbers with percentages for categorical variables. Continuous variables were compared using the Student t-test, and categorical variables were compared using the Chi-square test. Correlation analyses were performed using Pearson’s correlation coefficients to evaluate the relationship between CT scores and lung function or exercise capacity. We performed Cox proportional hazards analysis to identify risk factors for mortality in patients with HP, and variables with a *p*-value of <0.1 in the unadjusted analysis were included in the multivariable analysis with backward stepwise elimination. The follow-up time was calculated from the date of the initial HRCT to the date of death or time of censoring (date of vital status ascertainment: 04 February 2020). The Kaplan–Meier survival analysis and log-rank test were used for survival analysis. To compare the performance of each variable for predicting mortality, receiver operating characteristic (ROC) curve analysis for 5-year mortality was performed, and the optimal cut-off value was estimated by the Youden index method (30). The predicting performance of various combined models was compared by concordance statistics (C-statistics) proposed by Kang et al. (31). Statistical significance was defined as a *p-*value <0.05. All statistical analyses were performed using the SPSS software (version 21.0; IBM Corporation, Somers, NY, United States), MedCalc Statistical Software (version 12.7.5; MedCalc Software bvba, Ostend, Belgium), or R software (version 4.1.0, The R Foundation for Statistical Computing, Vienna, Austria; URL http://www.R-project.org/).

## Results

### Baseline characteristics

The mean age of all patients was 58.9 years, 60.4% were females, and 85.1% had a history of exposure to suspected inhalational antigens (Table 1). During the follow-up (median: 55.5 months, IQR: 37.0–89.0 months), 20 (19.8%) patients experienced acute exacerbation and 42 patients (41.6%) died. The 1-, 3-and 5-year mortality rates were 3.9, 16.8, and 32.7%, respectively. Among them, 25 patients showed respiratory-related death. The non-survivors were older, had lower lung function (FVC, DLco, and TLC) and poorer exercise capacity (distance and the lowest oxygen saturation on pulse oximetry [SpO_2_] during 6MWT) than the survivors (Table 1).

**Table 1:** Comparison of baseline characteristics between the non-survivors and survivors among patients with fibrotic HP.

Characteristic	Total	Non-survivors	Survivors	*p*-value
Patient numbers	101	42	59	
Age, years	58.9 ± 10.8	61.8 ± 11.0	56.8 ± 10.6	0.022
Female sex	61 (60.4)	28 (66.7)	33 (55.9)	0.277
BMI, kg/m^2^	25.0 ± 4.1	24.3 ± 4.9	25.5 ± 3.4	0.133
Positive history of exposure*	86 (85.1)	34 (81.0)	52 (88.1)	0.457
Ever-smokers	38 (37.6)	13 (31.0)	25 (42.4)	0.243
*Pulmonary function test*				
FVC, % predicted	71.2 ± 17.7	66.6 ± 21.3	79.2 ± 14.3	0.047
DLco, % predicted	60.2 ± 16.9	55.4 ± 17.2	63.4 ± 16.1	0.025
TLC, % predicted	73.6 ± 13.4	69.9 ± 14.9	76.1 ± 11.7	0.030
*6-min walk test*				
Distance, meter	443.5 ± 88.1	407.7 ± 105.3	469.5 ± 62.3	0.001
SpO_2_ nadir, %	92.2 ± 4.3	90.8 ± 4.6	93.2 ± 3.8	0.007
*Treatment*				0.043
No treatment	10 (9.9)	1 (2.4)	9 (15.3)	
Steroid ± cytotoxic agents^†^	91 (90.1)	41 (97.6)	50 (84.7)	

Data are presented as mean ± standard deviation, or number (%), unless otherwise indicated. HP, hypersensitivity pneumonitis. BMI, body mass index. FVC, forced vital capacity._,_ DLco, diffusing capacity of the lung for carbon monoxide. TLC, total lung capacity. SpO_2_ nadir, lowest oxygen saturation during 6-min walk test; ^*^Suspected antigens were animal protein (*n* = 8), chemical agent (*n* = 18), microbes (*n* = 50) plant’s proteins (*n* = 7) enzymes (*n*=1), metal (*n*=1) and pharmaceutical agents (*n*=1). ^†^Cytotoxic agents include azathioprine, cyclosporine, or cyclophosphamide.

### HRCT parameters

The non-survivors had higher scores of reticulation, honeycombing, fibrosis and MA than the survivors (Table 2). However, there was no significant difference in the frequency of a UIP-like pattern between the non-survivors and survivors.

**Table 2:** Comparison of HRCT scores between the non-survivors and survivors patients with fibrotic HP.

	Total	Non-survivors	Survivors	*p*-value
Patient numbers	101	42	59	
Reticulation, %	14.8 ± 9.8	16.2 ± 9.1	11.6 ± 9.1	<0.001
Honeycombing,%	0.9 ± 2.5	1.7 ± 3.1	0.5 ± 1.9	0.026
GGO, %	10.7 ± 14.9	6.4 ± 10.3	13.7 ± 16.9	0.008
Consolidation, %	0.3 ± 1.8	0.03 ± 0.3	0.5 ± 2.4	0.136
Fibrosis, %	15.7 ± 10.6	20.9 ± 10.1	12.1 ± 9.5	<0.001
MA,%	12.7 ± 10.8	17.3 ± 9.9	9.4 ± 10.3	<0.001
UIP like pattern	56 (55.4)	28 (66.7)	28 (47.5)	0.056

Data are presented as mean ± standard deviation, or number (%), unless otherwise indicated; GGO, ground-glass opacity. HRCT, high-resolution computed tomography. MA, mosaic attenuation. UIP, usual interstitial pneumonia. Fibrosis score was defined as the sum of reticulation and honeycombing score. *P-value was below 0.05.

There were statistically significant negative correlations between fibrosis and reticulation scores and lung function (FVC, DLco, and TLC) and the lowest SpO_2_ during 6MWT (online supplementary Figure S1). In addition, honeycombing (*r* = −0.207, *p* = 0.038) and MA (*r* = −0.284, *p* = 0.004) scores were also negatively correlated with exercise capacity (the lowest SpO_2_ or distance during 6MWT); however, consolidation score showed no significant correlation with the results of pulmonary function test (PFT) and 6MWT (online Supplementary Table S1).

### Prognostic factors for the mortality

In the unadjusted Cox analysis, older age, lower lung function (FVC, DLco, and TLC) and the minimum SpO2 during 6MWT, higher scores of HRCT parameters (reticulation, honeycombing, fibrosis scores, and MA), and the presence of a UIP-like pattern on HRCT were significantly associated with mortality in patients with fibrotic HP (Table 3). In the multivariable analysis, reticulation (HR 1.038; 95% CI 1.004–1.074) and GGO (HR 0.949; 95% CI 0.915–0.984) were independent prognostic factors for mortality in patients with fibrotic HP, as well as age and DLco. Fibrosis score (HR 1.040; 95% CI 1.007–1.075) was also an independent predictor of reduced survival, as well as age (Table 4).

**Table 3:** Risk factor for mortality in patient with fibrotic HP using unadjusted Cox proportional hazards model.

		Unadjusted analysis	
Parameter	Hazard ratio	95% confidence interval	*p*-value
Age, years	1.059	1.023–1.097	0.001
Female sex	1.241	0.650–2.367	0.513
BMI, kg/m^2^	0.948	0.874–1.028	0.199
Positive history of exposure	0.657	0.302–1.429	0.289
Ever-smoker	0.680	0.352–1.316	0.253
*Pulmonary function test*			
FVC, % predicted	0.973	0.955–0.991	0.003
DLco, % predicted	0.971	0.953–0.989	0.002
TLC, % predicted	0.962	0.938–0.986	0.002
*6-min walk test*			
Distance, meter	0.994	0.991–0.997	<0.001
SpO_2_ nadir, %	0.894	0.837–0.954	0.001
*HRCT findings*			
Reticulation,%	1.059	1.033–1.086	<0.001
Honeycombing,%	1.159	1.067–1.259	<0.001
GGO,%	0.956	0.923–0.990	0.012
Fibrosis,%	1.065	1.039–1.091	<0.001
MA,%	1.043	1.018–1.068	0.001
Consolidation,%	0.733	0.391–1.377	0.334
UIP-like pattern	2.454	1.259–4.784	0.008

FVC, forced vital capacity. FEV_1_, Forced expiratory volume in 1 sec_,_ DLco, diffusing capacity of the lung for carbon monoxide; TLC, total lung capacity; SpO_2_ nadir, lowest oxygen saturation during 6-min walk test; GGO, ground glass opacity. MA, mosaic attenuation. UIP, usual interstitial pneumonia.

**Table 4:** Risk factor for mortality in patient with fibrotic HP using multivariable Cox proportional hazards model.

	HR (95% CI)					
Parameters	Model 1	Model 2	Model 3	Model 4	Model 5	Model 6
Age, years	1.060^*^(1.020–1.101)	1.059^*^(1.019–1.100)	1.070^*^(1.028–1.114)	1.058^*^(1.019–1.099)	1.053^*^(1.014–1.094)	1.058^*^(1.018–1.099)
FVC,% predicted	0.997 (0.974–1.020)	0.987 (0.963–1.010)	0.994 (0.971–1.016)	0.996 (0.974–1.019)	0.994 (0.971–1.017)	0.988 (0.965–1.011)
DLco, % predicted	0.967^*^(0.936–0.999)	0.966^*^(0.935–0.998)	0.956^*^(0.926–0.987)	0.969 (0.938–1.002)	0.961^*^(0.931–0.992)	0.964^*^(0.933–0.996)
SpO_2_ nadir, %	0.992 (0.912–1.079)	0.987 (0.908–1.074)	0.980 (0.901–1.067)	0.989 (0.909–1.077)	0.997 (0.918–1.083)	0.991 (0.913–1.076)
HRCT pattern						
Reticulation,%	1.038^*^(1.004–1.074)					
Honeycombing,%		1.092 (0.980–1.218)				
GGO,%			0.949^*^(0.915–0.984)			
Fibrosis,%				1.040^*^(1.007–1.075)		
MA,%					1.028 (0.994–1.064)	
UIP-like pattern						1.349 (0.669–2.720)

Variables with *p* < 0.1 in unadjusted Cox analysis were included in multivariable Cox analysis. TLC was excluded from the multivariate analysis due to significant correlation with FVC (*r* = 0.873, *p* < 0.001). Distance of 6-min walk test was excluded because it could be affected by other factor such as leg pain; FVC, forced vital capacity. DLco, diffusing capacity of the lung for carbon monoxide. SpO_2_ nadir, lowest oxygen saturation on pulse oximetry during 6-min walk test. GGO, ground glass opacity. MA, mosaic attenuation. UIP, usual interstitial pneumonia.

Fibrosis score was defined as the sum of reticulation and honeycombing score. *P-value was below 0.05.

### Performance of HRCT parameters

**Figure 1:**
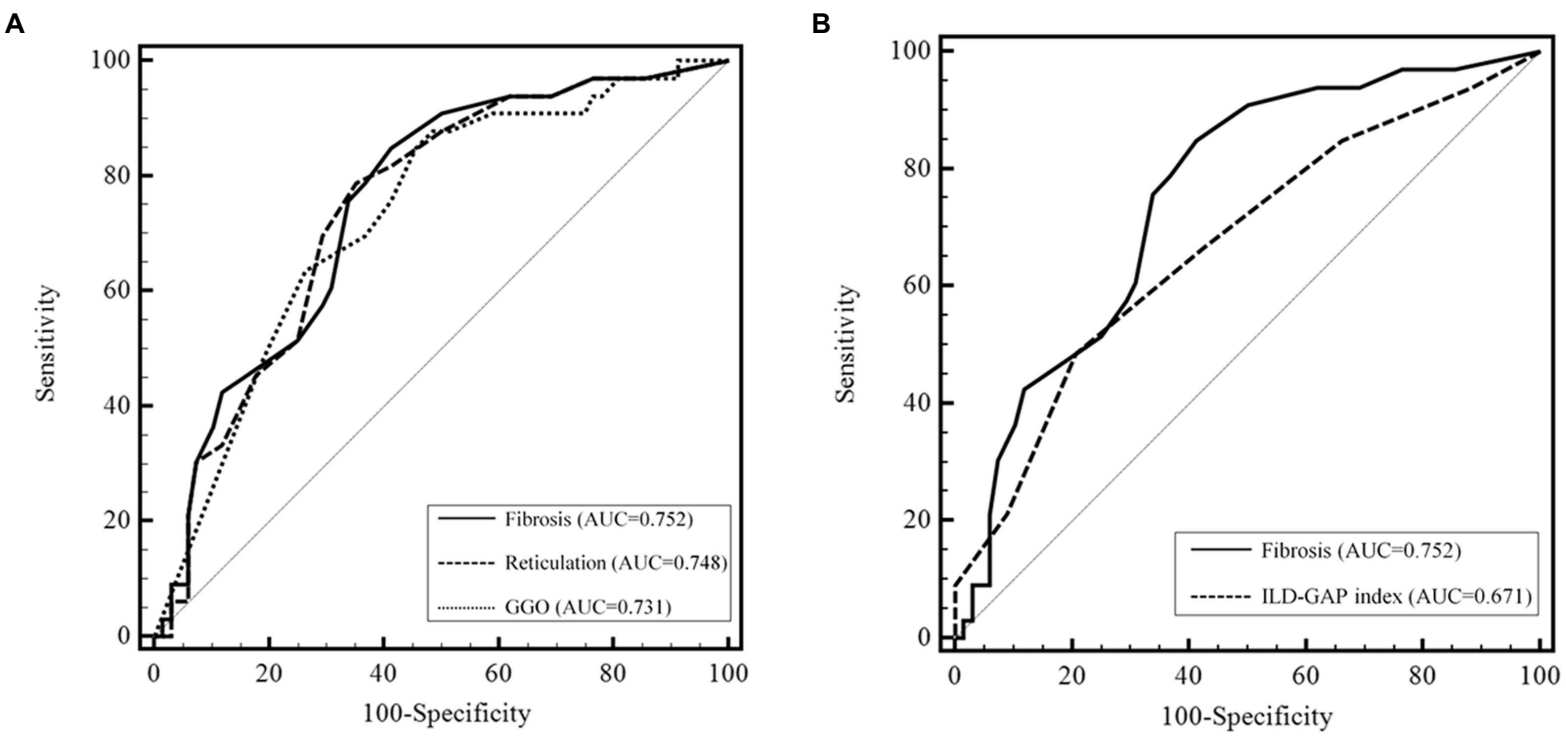
Predictive performance of CT scores for the 5 year mortality in patients with fibrotic HP. **(A)** Comparison of the predicting performance of the 5 year mortality among the CT scores. The area under the ROC curve was higher for the fibrosis score than for reticulation and GGO scores; however, the difference was not statistically significant (fibrosis, AUC = 0.752, 95% CI 0.656–0.833, *p* < 0.001; reticulation, AUC = 0.748, 95% CI 0.651–0.829, *p* < 0.001; GGO, AUC = 0.731, 95% CI 0.634–0.815, *p* < 0.001) (*p* = 0.758 and *p* = 0.697 between reticulation and fibrosis, GGO and fibrosis, respectively). **(B)** Comparison of the predicting performance of the 5 year mortality between fibrosis score and ILD-GAP index. CT, computed tomography. AUC, area under the curve. GGO, ground glass opacity.

**Figure 2:**
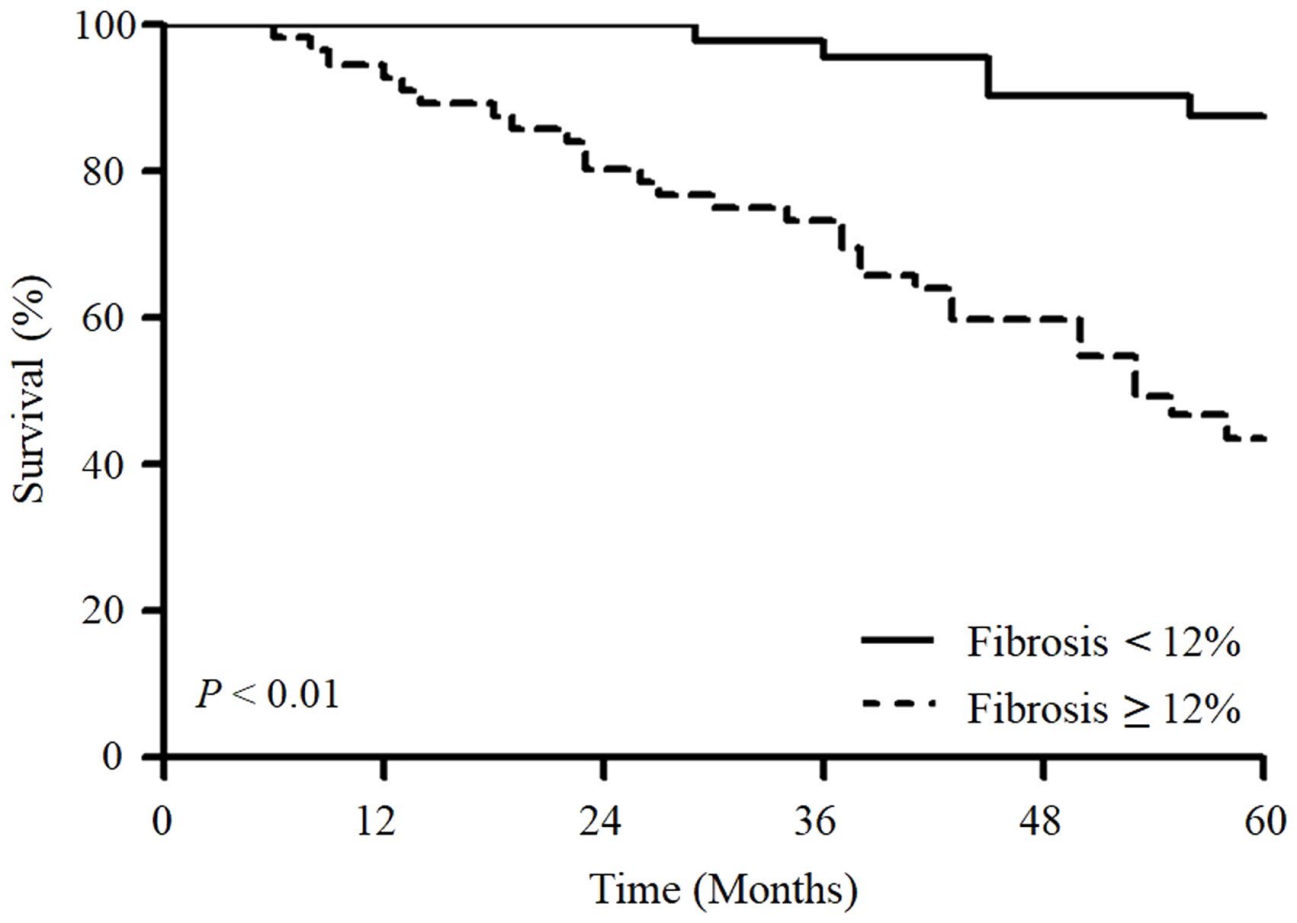
Kaplan–Meier survival analysis of overall survival according to fibrosis scores in patients with fibrotic HP. Patients with a fibrosis score above 12% had significantly worse survival than those with a score below 12% (*p* < 0.01).

In the ROC analysis, fibrosis scores had a higher area under the curve (AUC) than reticulation and GGO scores for predicting the 5-year mortality; however, the difference was not statistically significant (Figure 1A). In addition, the performance of the fibrosis score was comparable to the ILD-GAP index in predicting the 5-year mortality (Figure 1B). The optimal cut-off value of fibrosis score was 12.0% (sensitivity 84.9%, specificity 58.8%) for the 5-year mortality. Patients with high fibrosis score (≥12.0%) showed significantly worse prognosis (the mean survival time: 146.7 months [95% CI 129.5–164.1 months] vs. 58.3 months [95% CI 49.2–67.4 months], *p <* 0.01) than those with low fibrosis score (< 12%) (Figure 2).

To improve the predicting performance of the fibrosis score for the 5-year mortality, various risk prediction models, including clinical variables (age and DLco), were evaluated (online Supplementary Table S2); however, they did not outperform the model using the fibrosis score alone.

## Discussion

In this study, the radiologic fibrosis score was useful in predicting mortality in patients with fibrotic HP and showed a predictive performance comparable to that of the ILD-GAP model. Patients with high fibrosis scores (≥12.0%) had poorer survival outcome than those with low fibrosis scores. The radiologic fibrosis score was also an independent predictor for mortality after adjustment for age and physiologic parameters, and even without considering the clinical variables together, fibrosis score itself showed excellent predictive performance.

In this study, a higher fibrosis score on HRCT was associated with an increased mortality risk in patients with fibrotic HP. This result correlates with previous studies (4, 13, 14). Hanak et al. showed that a greater extent of fibrosis on HRCT resulted in higher overall mortality in 26 patients with fibrotic HP (21% vs. 83% in groups with <10% and > 40% of fibrosis extent, respectively) (14). In 132 patients of chronic HP, Chung et al. identified increasing reticulation as an independent predictor of reduced survival (HR 1.04; 95% CI 1.02–1.07) after adjustment for the presence of GGO and pulmonary artery/aorta ratio (4). In our study, reticulation was also significantly associated with mortality; however, the honeycombing score, one of the components of fibrotic features on HRCT, was not an independent prognostic factor in the multivariable analysis. This discrepancy might be due to the fact that the proportion of patients with radiologic honeycombing is not high, and most of them show low honeycombing scores. Similar to our finding, a previous study found that honeycombing had no statistically significant association with mortality after adjusting for GGO and pulmonary artery/aorta ratio in patients with chronic HP, even though subjects with honeycombing had worse survival than those without (log-rank test, *p* = 0.007) (4).

In our study, MA showed no significant association with mortality in patients with fibrotic HP after adjusting the clinical variables. However, in previous studies, MA was a predictor of better survival outcomes (5, 32). MA is assumed to be a secondary change due to small airway obstruction by obstructive bronchiolitis (33). Chung et al. showed that MA was associated with better survival in 110 patients with chronic HP (HR: 0.26; 95% CI: 0.07–0.97) after adjusting for age, smoking history, and FVC (5). Lima et al. also reported that in 103 patients with subacute/chronic HP, MA was an independent prognostic factor for mortality (HR: 0.05; 95% CI: 0.01–0.39) in the multivariate analysis along with age and oxygen saturation during 6MWT (32). These discordant findings may be due to the different characteristics of each cohort; previous studies included all kinds of HP (non-fibrotic and fibrotic HP) (5, 32), whereas our study included only fibrotic HP. Because MA is a common finding, particularly in non-fibrotic HP, MA may be associated with a relatively better prognosis (5, 14). It is unclear whether MA is associated with the progression or improvement of the fibrotic lesion in patients with fibrotic HP; however, Choe et al., in the study of the serial changes of CT images in patients with chronic HP, reported that the extent of MA remained constant over time, and were finally replaced by fibrosis while in progress (17).

In this study, a UIP-like pattern on HRCT showed no significant association with mortality in patients with fibrotic HP. The result of a previous study supports our findings; Chung et al., in a study of 132 patients with chronic HP, showed that a UIP pattern was not an independent predictor after adjusting for GGO, pulmonary artery/aorta ratio, and GAP index (4). Little is known about the prognostic role of a radiological UIP pattern in patients with fibrotic HP yet. Since UIP classification on chest CT has been predominantly used in IPF, the ability to adequately assess the prognosis of patients with HP may be limited (34–36). According to a previous study identifying the difference in UIP patterns between HP and IPF, HP patients with a UIP pattern had more frequent upper or middle lung dominance or extensive GGO than those with IPF (35). Therefore, in some studies that attempted to classify HRCT patterns in patients with ILD other than IPF, some modifications were applied to account for disease distributions or the presence of MA that was not consistent with a UIP pattern, as in our study (11, 29, 37). However, since there is no consensus on applying the UIP classification to HP, further studies are needed to confirm these results.

In the adjusted model including fibrosis score, only fibrosis score and age were independent risk factors for mortality in our study. Although some previous studies showed that reduced lung function was associated with poor survival in patients with chronic HP (6, 38, 39), these results were from the unadjusted analysis, and radiologic parameters were not considered together. In addition, a previous study, including 92 patients with chronic HP, identified that no individual PFT variables were independent predictors of mortality once HRCT patterns were taken into account for analysis, whereas traction bronchiectasis and honeycombing were associated with mortality (40). These results are comparable with our finding that image parameter was superior to physiologic parameters for predicting mortality in patients with fibrotic HP. These results can be explained by the fact that because HP is a kind of airway involvement disease, lung function abnormalities do not necessarily mean lung parenchymal change (41). Therefore, our results suggest that radiological parameters can be used as useful tools to overcome the limitations of physiologic parameters for predicting mortality in patients with HP.

This study has some limitations. First, it was a retrospective observational study conducted in a single center. Therefore, there might be selection bias leading to the non-generalizability of our findings. However, the baseline characteristics of our cohort were similar to those of other studies (9, 39). Second, the definition of a radiological UIP-like pattern used in this study was inconsistent with guidelines on the classification of IPF diagnostic. However, some modifications were inevitable to consider radiologic features of HP, such as upper or middle lung predominance or MA. Lastly, we did not consider comorbid conditions as confounders in the survival analysis. Because our study analyzed all-cause mortality as the primary outcome, comorbidities commonly found with fibrotic HP, such as pulmonary hypertension or heart failure, may have influenced the survival outcome (42).

In conclusions, our results suggest that a radiologic fibrosis score may be useful in predicting mortality, and high fibrosis score indicates poor prognosis in patients with fibrotic HP. Further prospective multicenter studies involving a larger population are needed to validate our findings.

## Data availability statement

The original contributions presented in the study are included in the article/Supplementary material, further inquiries can be directed to the corresponding author.

## Ethics statement

The studies involving human participants were reviewed and approved by Institutional Review Board of Asan Medical Center. Written informed consent for participation was not required for this study in accordance with the national legislation and the institutional requirements.

## Author contributions

JO, JK, and JWS: study concept and design, data collection, data analysis, interpretation, discussed the results, and reviewed the manuscript. JO and JWS: drafting of the manuscript. All authors contributed to the article and approved the submitted version.

## Funding

This study was supported by grants from the Basic Science Research Program (NRF-2022R1A2B5B02001602) and the Bio & Medical Technology Development Program (NRF-2022M3A9E4082647) of the National Research Foundation of Korea (NRF) funded by the Ministry of Science & ICT, Republic of Korea, and also supported by the National Institute of Health research project (2021ER120701) and by Korea Environment Industry & Technology Institute through Core Technology Development Project for Environmental Diseases Prevention and Management Program funded by Korea Ministry of Environment (ARQ202201450001), Republic of Korea.

## Conflict of interest

The authors declare that the research was conducted in the absence of any commercial or financial relationships that could be construed as a potential conflict of interest.

## Publisher’s note

All claims expressed in this article are solely those of the authors and do not necessarily represent those of their affiliated organizations, or those of the publisher, the editors and the reviewers. Any product that may be evaluated in this article, or claim that may be made by its manufacturer, is not guaranteed or endorsed by the publisher.
