# Supplementary material for: Fibrosis score predicts mortality in patients with fibrotic hypersensitivity pneumonitis
Source: Front Med (Lausanne). 2023 Mar 21;10:1131070. doi: 10.3389/fmed.2023.1131070 (PMC10070979; doi:10.3389/fmed.2023.1131070)
Supplement: Supplementary file 1 [file Data_Sheet_1.pdf]

Supplementary Material

Fibrosis score predicts mortality in patients with fibrotic hypersensitivity pneumonitis

Ju Hyun Oh[1], Jieun Kang[2], Jin Woo Song[3]

[1] Department of Pulmonary and Critical Care Medicine, Sanggye Paik Hospital, Inje University College of Medicine, Seoul, Republic of Korea;

[2] Division of Pulmonary and Critical Care Medicine, Department of Internal Medicine, Ilsan Paik Hospital, Inje University College of Medicine, Goyang-si, Gyeonggi-do, Republic of Korea;

[3]Department of Pulmonary and Critical Care Medicine, Asan Medical Center, University of Ulsan College of Medicine, Seoul, Republic of Korea

***Corresponding author:** Jin Woo Song, M.D., Ph.D.

Department of Pulmonary and Critical Care Medicine, Asan Medical Center, University of Ulsan College of Medicine, 88, Olympic-Ro 43-gil, Songpa-gu, Seoul 05505, Republic of Korea

Tel: 82-2-3010-3993

Fax: 82-2-3010-6968

E-mail: jwsongasan@gmail.com

Table S1. Correlation between HRCT scores and physiologic parameters

	Variables	Correlation coefficient	*P*-value
Honeycombing	FVC, % predicted	0.011	0.913
	TLC, % predicted	-0.030	0.775
	FEV1, % predicted	0.122	0.225
	DLco, % predicted	-0.175	0.093
	SpO2 nadir, %	-0.207	0.038
	Distance, meter	-0.191	0.057
Mosaic attenuation	FVC, % predicted	-0.133	0.187
	TLC, % predicted	-0.134	0.204
	FEV1, % predicted	-0.089	0.377
	DLco, % predicted	-0.095	0.367
	SpO2 nadir, %	-0.172	0.087
	Distance, meter	-0.284	0.004
Consolidation	FVC, % predicted	-0.012	0.902
	TLC, % predicted	0.055	0.605
	FEV1, % predicted	-0.090	0.371
	DLco, % predicted	-0.113	0.282
	SpO2 nadir, %	-0.084	0.404
	Distance, meter	-0.192	0.055

FVC, forced vital capacity; FEV_1, Forced expiratory volume in one second; DLco, diffusing capacity of the lung for carbon monoxide; TLC, total lung capacity; SpO_2 nadir, The lowest oxygen saturation during the 6-minute walk test

Table S2. Comparison of the performance of risk prediction models for mortality in patients with fibrotic HP

Models	C-index (95% CI)	*P*-value
(1) Fibrosis score	0.726 (0.624-0.814)	reference
(2) Fibrosis score + age	0.720 (0.617-0.809)	0.952
(3) Fibrosis score + DLco	0.753 (0.652-0.837)	0.331
(4) Fibrosis score + age + DLco	0.772 (0.674-0.853)	0.248

CI, confidence interval; HP, hypersensitivity pneumonia; DLco, diffusing capacity of the lung for carbon monoxide;

Figure S1. Correlation between HRCT scores and physiologic parameters

(A) Correlation between fibrosis score and physiologic parameters (PFT and 6MWT), (B) Correlation between reticulation score and physiologic parameters (PFT and 6MWT); HRCT, high-resolution computed tomography; PFT, pulmonary function test; 6MWT, 6-minute walk test; SpO_2 nadir, lowest oxygen saturation during the 6-minute walk test

(A)

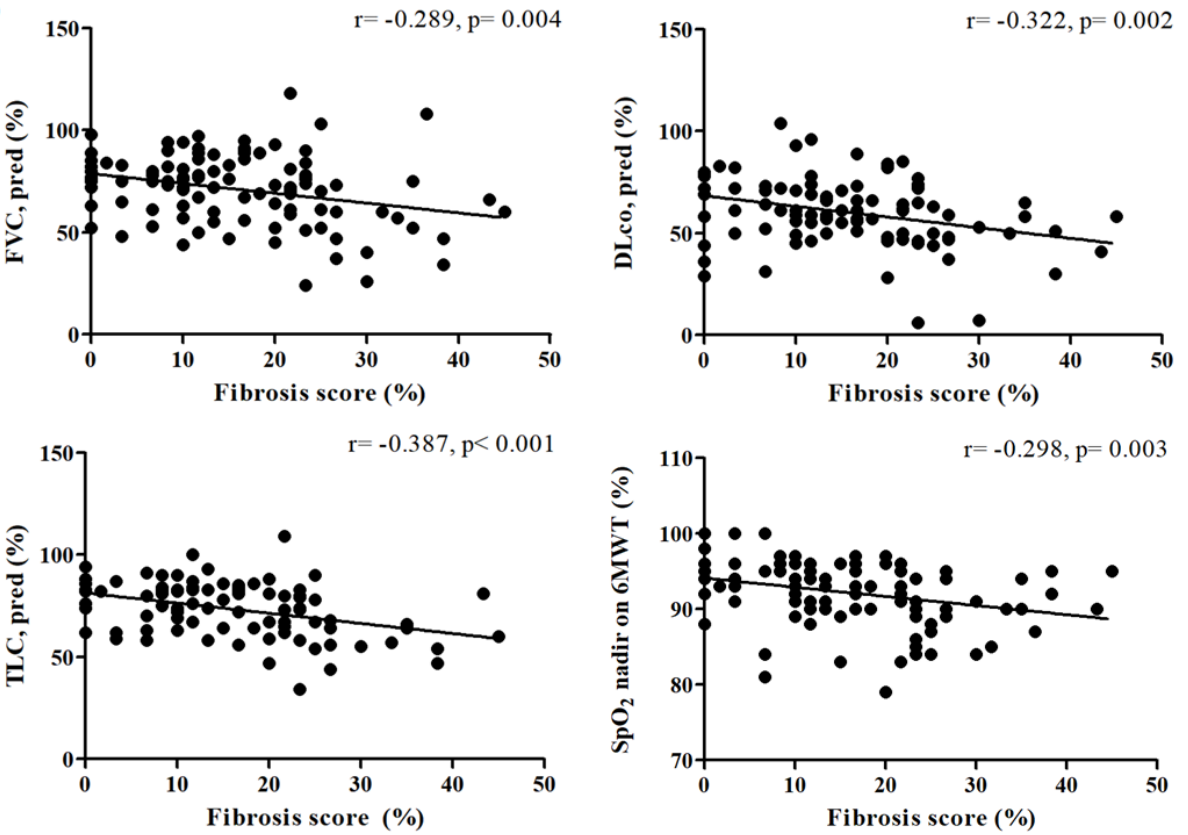
r= -0.289, p= 0.004
FVC, pred (%)
Fibrosis score (%)
r= -0.322, p= 0.002
DLco, pred (%)
Fibrosis score (%)
r= -0.387, p< 0.001
TLC, pred (%)
Fibrosis score (%)
r= -0.298, p= 0.003
SpO2 nadir on 6MWT (%)
Fibrosis score (%)

(B)

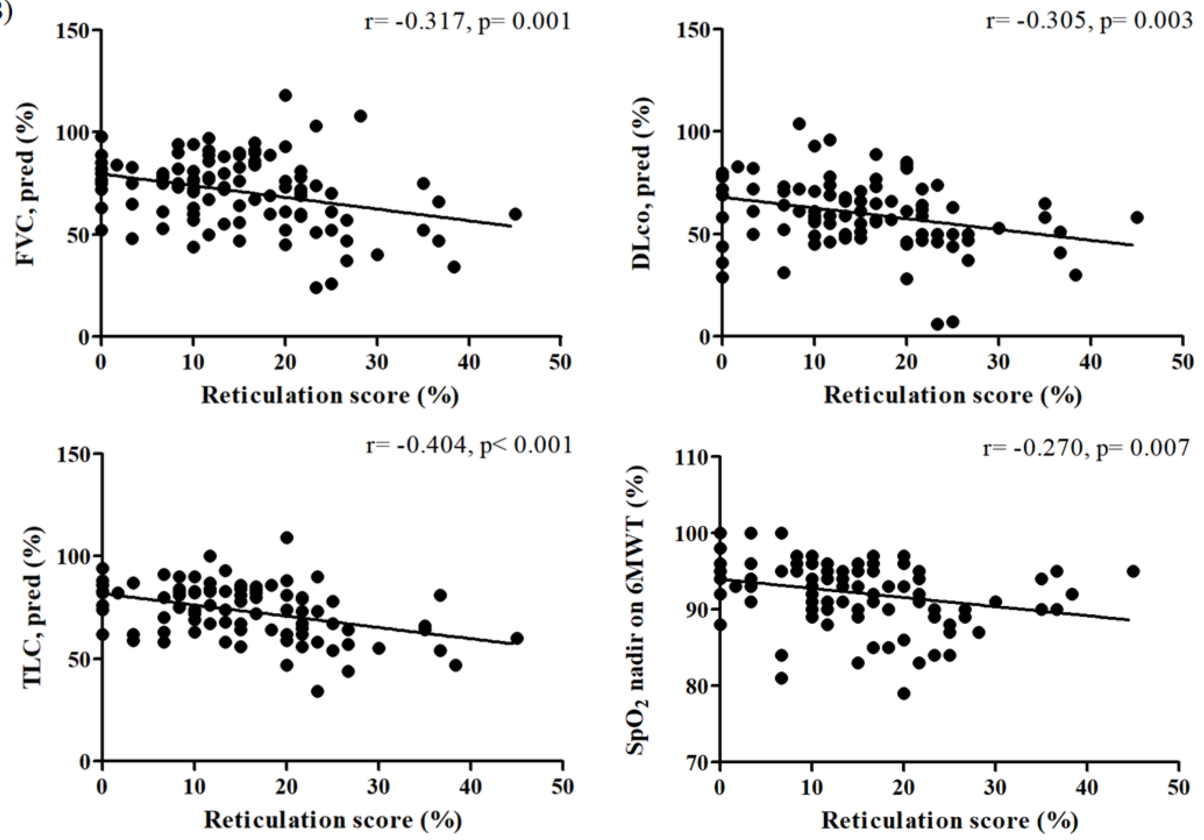
r= -0.317, p= 0.001
FVC, pred (%)
Reticulation score (%)
r= -0.305, p= 0.003
DLco, pred (%)
Reticulation score (%)
r= -0.404, p< 0.001
TLC, pred (%)
Reticulation score (%)
r= -0.270, p= 0.007
SpO2 nadir on 6MWT (%)
Reticulation score (%)
